# Spillover Effects of Loss of Control on Risky Decision-Making

**DOI:** 10.1371/journal.pone.0150470

**Published:** 2016-03-01

**Authors:** Birgit M. Beisswingert, Keshun Zhang, Thomas Goetz, Urs Fischbacher

**Affiliations:** 1 Department of Empirical Educational Research, University of Konstanz, Konstanz, Germany; 2 Graduate School of Decision Sciences, University of Konstanz, Konstanz, Germany; 3 Department of Empirical Educational Research, Thurgau University of Teacher Education, Kreuzlingen, Switzerland; 4 Department of Economics, University of Konstanz, Konstanz, Germany; 5 Thurgau Institute of Economics, Kreuzlingen, Switzerland; University of Reading, UNITED KINGDOM

## Abstract

Decision making in risky situations is frequently required in our everyday lives and has been shown to be influenced by various factors, some of which are independent of the risk context. Based on previous findings and theories about the central role of perceptions of control and their impact on subsequent settings, spillover effects of subjective loss of control on risky decision-making are assumed. After developing an innovative experimental paradigm for inducing loss of control, its hypothesized effects on risky decision-making are investigated. Partially supporting the hypotheses, results demonstrated no increased levels of risk perceptions but decreased risk-taking behavior following experiences of loss of control. Thus, this study makes a methodological contribution by proposing a newly developed experimental paradigm facilitating further research on the effects of subjective loss of control, and additionally provides partial evidence for the spillover effects of loss of control experiences on risky decision-making.

## Introduction

Risky environments are part of people’s everyday lives. We are not only exposed to risky contexts, but are often required to make immediate decisions on how to respond to these hazards and on which risks to take. Some everyday decisions are characterized by some degree of risk, such as deciding on insurance contracts or investment options regarding our financial concerns. These decisions, including the assessment of the probability of the different outcomes, are widely regarded as a result of complex deliberating processes. There is considerable heterogeneity in risk taking [1], and preceding events as well as characteristics of the actual situation impact the decision-making processes, among which subjective perceptions of personal control play an important role [2]. Perceptions of control can be defined as an individual’s belief about the extent to which he or she can predict or influence events [3]. These perceptions result from the belief that there is a contingent connection between one’s behaviors and the resulting outcomes and the belief that one is able to perform the required behavior successfully [4]. Perceptions of control might influence decision under risk because they can shape the perceived riskiness of a decision or the willingness to take the risk. This question is relevant because, people might frequently experience a lack or loss of control in a world of rapid changes, increasing complexity and novel challenges in their everyday lives (e.g., [5]).

Thus, being able to deal with experiences of loss of control appropriately and to understand how they affect everyday decisions, such as choices made under risk conditions, carries practical pertinence. However, so far research on the relationship between control experiences and risky decision-making is scarce. This may partly be due to the fact that suitable experimental paradigms to systematically examine the effects of perceived control are lacking. Thus, in this study we aimed to overcome this shortage by proposing a newly developed experimental paradigm for inducing subjective loss of control. We also aimed to apply our paradigm to investigate the effects of prior subjective loss of control experiences on subsequent risky decision-making, specifically, on risk perception and risk-taking behavior in a risky setting.

### Objective and Subjective Aspects of Risky Decision-Making

We define decision risk as “the extent to which there is uncertainty about whether potentially significant and/or disappointing outcomes of decisions will be realized” [6, 7], which means that the possible outcomes occur with some probability. Thus, the objective extent of the risk involved in a given hazard may be defined using a combination of probabilities and outcomes [2]. However, there are notable differences in people’s ways of dealing with risky situations, even when they face comparable hazards with analogous threat potential and probability, and therefore objectively identical risk conditions [1, 8]. These individual differences can stem from two sources. First, they might be due to the fact that “individuals respond according to their perception of risk and not according to an objective risk level or the scientific assessment of risk” ([2], p. 60). Second, even if perceptions of risk are similar, risky behavior may differ depending on an individual’s willingness to bear risks (e.g., [6]). When aiming to understand people’s risky decision-making, it is therefore reasonable to take “objective” risk conditions, individual’s “subjective” risk perceptions (cognition) as well as the actual risk-taking decision (behavior) into account.

### Spillover Effects of Control Perceptions on Risky Decision-Making

The phenomenon that people’s experiences in one domain impact their cognitions, emotions and behavior in another–even objectively unrelated–context is a widely accepted and investigated occurrence known as “spillover effect” (e.g., [9, 10]). Spillover effects on subsequent risky decision-making may be especially important to consider in relation to subjective experiences of personal control. Their substantial role in the deliberation process for decisions and behavior in risky situations has been supported empirically (cf. [2]); and research on illusions of control has added evidence regarding the impact of control perceptions in chance situations ([11], for a review see [12]). An especially strong association between people’s perceived control and their risk perceptions has been identified with both variables shown to be negatively correlated (cf. [13, 14]). Perceiving oneself “in control” can cause underestimations of riskiness and encourage more risky behavior. Conversely, feelings of loss of control might be accompanied by a sense of threat and the motivation to restore perceptions of control [15], thus suggesting perceptions of high riskiness and refusal to expose oneself to even more insecurity may cause rather risk-averse behavior. In line with this negative correlational relationship perceived control is sometimes even treated as an indicator of risk perception (cf. [16]).

Based on this prior line of research, we expect that the experience of subjective loss of control will affect subsequent risk-related cognitions (i.e., increased risk perceptions) and behavioral decision making (i.e., decreased actual risk taking) due to spillover effects. This proposition is in line with three different well-established theoretical models, which can be classified to either (a) emphasize the consequences of perceived control or (b) be concerned with the risk-related perception and decision-making process. Despite their difference in focus, all three research lines address the assumed impact of control experiences on risky decision-making:

The Theory of Learned Helplessness [17, 18], belonging to the former category, represents one of the most prominent frameworks addressing spillover effects and is the first theoretical model considered. It explains the empirically well illustrated emotional, cognitive, motivational and behavioral impairments that follow perceived lack of control, even in unrelated subsequent contexts. Following this model, our proposed effects on subsequent risk-related judgments and behavior can be regarded as one of the consequences of a perceived lack of personal control. Second, when turning to the latter category of models, assumed spillover effects can also be drawn from the risk-as-feeling-approach [19]. Consistent with this approach it is proposed that an individual’s previous experiences may also impact the decision-making process. Third, changes in risk-taking behavior following subjective experiences of uncontrollability may be due to current impairments of risk perception. For example, Tversky and Kahneman [20, 21] suggested that deviations in subjective judgments of probability are caused by the mental availability of appropriate events (intuitive “availability bias”).

### Previous Research on Perceived Control and Risky Decision-Making

Despite the fact that from a theoretical point of view the relationship between preceding lack of control experiences and risk-related decisions appears plausible, there is little empirical research on the supposed relationship between perceived control and subsequent risk-related cognitions and behavior. In particular, experimental studies which systematically explore the effects of variations in perceived control (or related constructs) on risk decisions are scarce. As one of the very few exceptions, Rivers and Arvai [22] investigated the effect of chronic losses on decision making under risk using an experimental laboratory design. They examined how risk-taking behavior within a single answer lottery would be affected by taking part in a gambling game of seven rounds in which the participants–despite being told that the probability of winnings would be 0.7 on average–through preinstalled winning probabilities either experienced chronic losses (winning probability *p* = 0.1), random outcomes (*p* = 0.5) or chronic wins (*p* = 0.9). Results demonstrated that the participants who had suffered unexpected chronic losses acted significantly more risk averse in a subsequent lottery game. Thus, Rivers and Arvai’s study provides valuable support for the hypothesized relationship between perceived control and subsequent risk-related cognitions and behavior as they found that decreased risk-taking behavior in a subsequent lottery followed their experimental manipulation of chronic losses within a preceding gambling game. However, the study faces a few methodological shortcomings. First, despite arguing on the basis of the Theory of Learned Helplessness, the subjectively perceived extent of control was not explicitly measured. Second, they used hypothetical money and installed deviant probabilities for winning in the manipulated conditions without the participants’ knowledge and therefore produced “unnaturally” occurring losses or wins. Further, the experimental design used deception. Other previously used classical experimental designs (e.g., [23–25]) used deception when inducing subjective loss of control as well. This prevents these designs from being applied in a more widespread manner because participants who experience deception may no longer respond in a natural way to the incentives. This is why the no deception standard is widely accepted in experimental economics (cf. [26]).

Although the psychology literature found robust effects of illusion of control on risky decision-making [27], the economic literature found little or no evidence of illusion of control (e.g., [28, 29]). As argued by Filippin and Crosetto [30], there are two possible reasons for the dissonance in findings. One possible explanation is that illusion of control could be observed in hypothetical choices, but the evidence usually disappears when monetary incentives are adopted [30, 31]. Another possible reason is that evidence of illusion of control from the economic literature is usually induced by manipulating the probability of success directly in the resolution of uncertainty. While there is another form of illusory control that focus on more concrete aspects of the choice itself in the psychological literature, for example, subjects being allowed to choose their lottery ticket rather than being assigned one [11]. These two types of illusion of control in the two disciplines are distinct from each other, which might cause the striking differences in findings. Therefore, new experimental paradigms are needed to prove the effect of illusion of control on risky decision-making, in which incentivized setting should be adopted and participants be allowed to control over the choice of uncertainty.

That is why new experimental designs meeting the guidelines and ethical criteria of experimental economics (e.g., no deception, incentivized setting) are much needed in order to investigate the risk-related effects of subjective loss of control.

## Research Aims and Hypotheses

The objectives of our set of studies were twofold: The first step was to develop and pilot an appropriate experimental paradigm for inducing subjective loss of control (Pilot Experimental Studies 1 and 2) in an incentivized setting. In order to prove the efficacy of this newly developed experimental paradigm, the second objective then aimed at applying it to investigate the spillover effects of subjective loss of control on risky decision-making (Main Experimental Studies 1 and 2). Risky decision-making was assessed by two separate indicators: risk perception as a cognitive measure (Main Experimental Study 1), and risk taking as a behavioral measure (Main Experimental Study 2). We expected both measures to be affected by prior experiences of subjective loss of control. Specifically, we were interested in testing two hypotheses.

Hypothesis 1: Due to spillover effects, prior subjective loss of control experiences are assumed to result in increased estimations of risk in a subsequent risky situation (cf. Main Experimental Study 1).

Hypothesis 2: Due to spillover effects, prior subjective loss of control experiences are assumed to result in decreased risk-taking behavior in a subsequent risky setting (cf. Main Experimental Study 2).

### Ethical Statement

The present study was conducted in compliance with the ethical standards expressed in the WMA Declaration of Helsinki. The study has been approved and all study procedures have been deemed appropriate by the Institutional Review Board of the University of Konstanz (S1 Appendix). Prior to participation, participants were informed about the precise contents of the study with paper instructions (goals of the research, duration, procedure, profits and anonymity in the experimental processes as well as in the data analyses) before they started their experiments. Voluntary participation was compensated by a fixed participation fee supplemented by payment according to the subject’s performance in a problem-solving and a classic economic game. This procedure was in compliance with ethical standards provided by the Federation of German Psychologists Association and the American Psychological Association. Guidelines provided by these institutions state that formal informed consent is not obligatory when no potential harm or distress is to be expected and/or when normal educational practices are followed as a goal of the research. Furthermore, all identifiers that could link individual participants to their results were not included in the experimental processes as well as in the data analyses, therefore, all analyses were conducted on anonymous data.

## Pilot Experimental Study 1

### Aims

Facing a lack of appropriate designs, the aim of this study was to pretest several potential components and decide on their inclusion in a new experimental design developed to induce subjective loss of control. With “loss” implying a change in possession, the experimental design was intended to make the participants perceive a gradual alteration in the extent of control. To this end, a computer-based problem solving task was chosen and task difficulty was manipulated to gradually increase in order to induce subjective loss of control. This first pilot study aimed at pretesting various aspects of the problem-solving task in terms of their objective and perceived difficulty in order to select the most appropriate components for the experimental design.

### Method

#### Overview: Computer game

**Fig 1:**
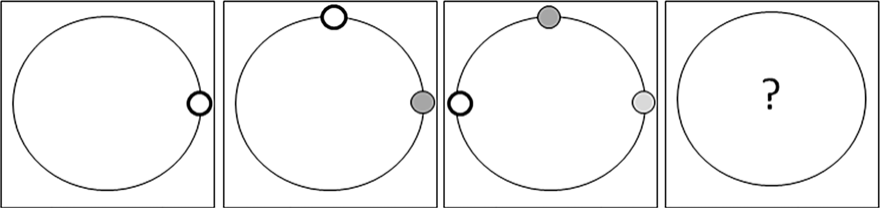
A sample schematic representation for a low difficulty pattern in the computer-based problem-solving task. Participants are asked to continuously indicate the assumed next position of the little white circle while its previous positions fade from dark grey to lighter shades of grey.

The problem-solving task consisted of an incentivized computer game in which the participants had to predict by mouse click where an object would be displayed on a circle by recognizing the systematic pattern underlying the previously displayed objects (e.g., clockwise or equidistant, for an example see Fig 1). As the experimental paradigm was supposed to consist of an experimental-control group pre-post-design with both conditions, including four rounds of the game-based problem solving task, this pilot study pretested 30 potential patterns. The goal was to select the most appropriate 12 patterns for inclusion in the subsequent experiments: eight patterns with low difficulty (experimental and control group baseline part as well as control group manipulation part) and four patterns with increasing difficulty (experimental group manipulation part). This computer-based paradigm was developed using z-Tree [32].

#### Participants and data collection

N = 34 students (74% female) with an average age of M = 22.29 years (SD = 5.23, range: 19–45) voluntarily participated in this study. Participants were compensated by a fixed show-up (9 €) fee plus payment according to their individual performance in the problem-solving game (theoretical range: 0–8 €).

In a one-factorial within-subjects design the participants played 30 rounds of the problem-solving task with each of them using a different underlying pattern. The patterns were determined by the angular distances of the subsequently displayed objects from the position of the object in the preceding display. Thus, the task of recognizing these sequences is similar to solving a continuing number series or pattern–a frequently used nonverbal reasoning task in common tests of intelligence (for example see the K-ABC-II, CogAT6, CFT-20-R, [33–35]). The 30 items were created specifically for this study and they differed from each other only with respect to the underlying patterns which determined their task difficulty. They were tested in order to choose the 12 patterns most appropriate for the experimental design in order to induce difficulty-related subjective loss of control. Thus, objective and subjective difficulty indicators, as well as their correspondence, served as criteria for choosing the 12 patterns ultimately selected for the experimental design. The order of the patterns was randomized between the participants. Each sequence was repeated until the maximum number of 25 predictions was reached (25 time intervals). In order to analyze the participants’ predictions and the objective difficulty of each pattern, the participants’ performance in the 4^th^ through 25^th^ time intervals were used.

#### Variables and study measures

The participants’ performance was assessed by the measured angular deviation of the distance between the predicted position by the participant and the position where the object was actually displayed on the circle. Thus, theoretically, mean performance could range between 0° (perfect prediction) and 180° (worst prediction) for each participant and pattern. The average performance of all participants served as an indicator of objective difficulty of the pretested patterns.

Following each of the 30 patterns the participants were asked to rate their experienced, thus subjective, perception of the difficulty level with respect to the previous round (“How difficult was the previous round for you?”) on a seven-point-rating-scale ranging from 0 *extremely easy* to 6 *extremely difficult*. The subjective ratings of perceived difficulty were analyzed and compared with the participants’ objective performance in order to select 12 (eight low difficulty and four increasing difficulty patterns) of the 30 pretested patterns for the experimental design.

### Results

The average subjective difficulty of the 30 patterns was *M* = 2.25 (*SD* = 1.33, range: 0.09–5.12), the average angular deviation as an indicator of objective difficulty was *M* = 27.55° (*SD* = 13.52°, range: 7.04° - 58.34°; theoretical range: 0° - 180°). The patterns’ subjectively rated difficulty, the objective difficulty and the correspondence between the two difficulty measures were used as criteria for the selection.

Descriptive statistics of the 12 selected patterns with respect to the subjective and objective difficulties and their Pearson correlations are displayed in Table 1. For details on the angular sequences underlying the patterns see the S2 Appendix. Patterns 1 to 8 were selected to represent low complexity. Although both the subjective and objective difficulty indicators of patterns 5 to 8 tended to be slightly higher, *t*-tests revealed no significant differences in the subjectively rated difficulty between each pair of subsequent patterns from 2 to 8, thus indicating no distinguishable increase in perceived difficulty. Both the subjective and objective difficulties of patterns 1 to 8 were below the average of all 30 pretested rounds.

**Table 1:** Descriptive Statistics and Correlations Between the Subjective and Objective Difficulties of the 12 Selected Patterns.

Category of Difficulty	Selected Pattern	Subjective Difficulty		Objective Difficulty		*r* (Subjective—Objective Difficulty)
		*M*	*SD*	*M*	*SD*	
Low	1	1.18	0.94	20.00	8.48	.49**
	2	1.47	1.11	16.77	6.83	.60***
	3	1.79	1.32	19.32	9.13	.35*
	4	1.41	1.23	19.67	9.27	.69***
	5	1.91	1.42	31.27	12.02	.75***
	6	1.88	0.95	20.58	5.73	.54**
	7	1.41	1.26	16.61	8.58	.42*
	8	1.59	1.54	24.42	14.60	.87***
Increasing	9	2.26	1.52	30.33	11.93	.77***
	10	3.44	1.86	36.13	14.82	.88***
	11	4.29	1.19	52.56	9.59	.48**
	12	5.12	0.88	53.08	10.45	.71***

*Note*. *N* = 34. Patterns 1 to 8 represent the low difficulty patterns, whereas patterns 9 to 12 represent increasing difficulty patterns.

*p < .05.

**p < .01.

***p < .001.

In contrast, for patterns 9 to 12 both the subjective and objective difficulties were above average (subjective difficulty: *M* = [2.26, 5.12]; average angular deviation as a measure of objective difficulty: *M* = [30.33°, 53.08°]). Additionally, both the subjective and objective difficulty indicators significantly increased between each pair of subsequent patterns 9 to 12, indicating the proposed rise in difficulty.

With respect to all the 12 selected patterns the Pearson correlations between the subjective and objective difficulty measures ranged between *r* = .35, *p* = .044 (pattern 3), and *r* = .88, *p* < .001 (pattern 10), *Mdn* = .65, representing medium to high relationships according to Cohen [36].

### Discussion

The results of the pretested patterns allowed a selection of 12 (eight low and four middle to high difficulty) patterns to be included in the experimental design. Patterns 1–4 were selected for the low difficulty baseline portion; similarly, patterns 5 to 8 were chosen for the control group’s manipulation section for which the difficulty has to be kept at a relatively stable, low level. Finally, patterns 9–12 were chosen for the experimental group’s manipulation section for which difficulty is intended to increase in order to induce subjective loss of control due to increasing task difficulty.

While the selection criteria were based on both subjective and objective difficulty indicators, a remarkable correspondence between the individuals’ perception (subjective difficulty) and performance in these tasks (objective difficulty) was observed, especially for the more difficult patterns. Thus, despite the fact that this study is mainly focusing on the effects of subjectively perceived extents of control, their correspondence with the objectively given conditions is very satisfying and the selection of patterns for the experimental design based on this pilot experimental study can be considered valid.

## Pilot Experimental Study 2

### Aims

The objective of Pilot Experimental Study 2 was to investigate whether the increase in objective task difficulty would impact participants’ subjectively perceived extent of control. Therefore, the experimental design included the previously tested and selected patterns of varying difficulty. By assessing the participants’ subjective control ratings, the question to be explored was whether the objectively produced decrease in control (due to increasing task difficulty) is subjectively perceived as a loss of control.

### Method

#### Participants and data collection

N = 42 students (50% female) recruited using the online recruiting system ORSEE [37] participated in Pilot Experimental Study 2 and had an average age of M = 22.00 years (SD = 2.45, range: 19–30). The subjects participated voluntarily and were compensated by a fixed participation fee (9 €) plus payment according to their individual performance in the problem-solving game (maximum profit: 1 € per round). The participants were randomly assigned to the treatment conditions with n = 20 (50% female) in the experimental (EG) and n = 22 (50% female) in the control group (CG).

**Fig 2:**
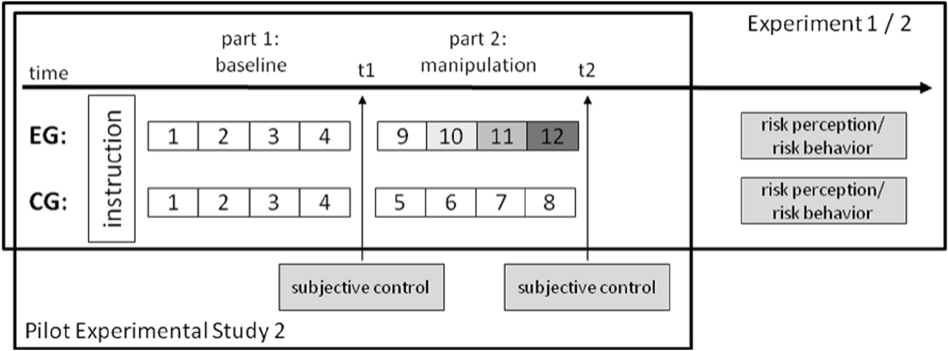
Design of Pilot Experimental Study 2 and Main Experimental Studies 1 and 2. Pilot Experimental Study 2 tested the experimental computer-based problem-solving paradigm that included 12 different task patterns for inducing difficulty-related loss of control. In a one-factor pre-post design the experimental (EG) and control groups (CG) were compared using subjective control questionnaires following the baseline (t1) and manipulation (t2) parts of the experiment. Main Experimental Studies 1 and 2 applied the same computer-based problem-solving paradigm to investigate the effects of loss of control on risk perception and risk behavior, respectively.

The experimental study consisted of a one-factor pre-post design with questionnaires following the baseline and manipulation sections (cf. Fig 2) to allow for both between- and within-subject comparisons. Following an instruction phase which included a comprehension test at the end to ensure appropriate understanding of the task, both the experimental and control group played eight rounds of the computer-game based problem-solving task. The first four rounds represented the baseline section in which both groups were presumed to experience subjective control (patterns 1–4), while the second four rounds constituted the manipulation section. In this manipulation section different patterns were used for the two groups. While the control group played patterns 5 to 8 whose difficulty remained relatively stable on a low level, the experimental group played patterns 9 to 12 which represented increasing task difficulty.

Between the two sections (t1) and at the end of the manipulation section (t2) the participants answered a questionnaire assessing their subjective perceptions of control. This served as a dependent variable in order to test whether the increase in task difficulty during the manipulation section of the EG was subjectively perceived as a loss of control. The participants were asked to answer these questionnaires concerning the previously played pattern, thus following pattern 4 for all participants and following patterns 12 or 8, respectively, depending on whether they were part of the experimental or control section. Socio-demographic and several potentially relevant trait variables were assessed during a separate follow-up which each participant attended within about two weeks following the experimental part of the study. Participants were compensated by a fixed fee 5 € (part of the total fixed participation fee 9 €) in the follow-up experiment.

#### Variables and study measures

Following the baseline (t1) and manipulation sections (t2), the participants were asked to indicate their subjective perceptions of control over their outcomes with respect to the previously played round. The items are based on the Academic Control Scale [38] and were adapted to the computer-game context (e.g., “I could completely determine my outcomes.”; “I myself could determine the accuracy of the target position on the screen.”). Using a seven-point-rating-scale ranging from *0 completely disagree to 6 completely agree*, the two-item-measure had an internal consistency of α = .62 (t1) and α = .66 (t2). The correlation between the two items was *r* = .45, *p* = .003 (t1) and *r* = .50, p = .001 (t2).

The participants’ performance during the manipulation section, as measured by the average angular distance between the participants’ clicking position and the target location, served as a manipulation check to ensure that increasing difficulty impacted performance.

Two sets of potentially relevant traits and socio-demographic variables were assessed in a separate follow-up which the participants attended approximately within the two weeks following the experiment:

Three subtests (“number series”, “figure selection” and “cubes”; 20 items each) of a prominent and widely used German intelligence structure test (I-S-T 200-R, [39], English version, [40]) were assessed as indicators of numeral and figural nonverbal reasoning.

A German version of Levenson’s [41] Internality, Powerful Others and Chance-Scales (IPC) was applied as a measure of internal versus external locus of control [42]. This measure consisted of the three 8-items-subscales: internal control orientation (I; e.g., “I can pretty much determine what will happen in my life”), powerful other external control orientation (P; e.g., “I feel like what happens in my life is mostly determined by powerful others”) and chance control orientation (C; e.g., “To a great extent, my life is controlled by accidental happenings”). Participants were asked to indicate their agreement with the 24 statements on a 6-point rating scale ranging from -3 *strongly disagree* to +3 *strongly agree*. Due to reliability concerns we joined the subscales P and C to a compound 16-items external control orientation subscale and conducted the data analyses accordingly. Cronbach’s alpha was α = .62 for subscale (I) and α = .69 for the 16-items external orientation subscale (P and C, Results are presented according to the data analyses using the compound 16-items external control orientation subscale. However, the same pattern of results occurs when using subscales P and C separately as compared to the compound subscale. Reliabilities of the separate 8-items-subscales were α = .65 (P) and α = .60 (C).).

Participants’ sex and age, as well as information on their chosen subjects of study, mother languages and subjective ratings of their experiences with computer games were assessed through a questionnaire.

### Results

#### Trait variables

*t*-tests for equivalence revealed no significant differences between the treatment groups with respect to nonverbal reasoning indicators and general control beliefs. However, the participants of the experimental group turned out to be significantly older than the members of the control group, EG: *M* = 23.10, *SD* = 2.65 versus CG: *M* = 21.00, *SD* = 1.77; *t*(40) = 3.04, *p* = .004; *d* = 0.94. For this reason all analyses were run with and without treating age as a covariate with negligible differences in the findings between the two methods. Thus, the subsequent results are presented with age included as a covariate in ANCOVA analyses.

#### Manipulation check

In accordance with our expectations, the participants’ performance in the problem-solving task during the manipulation section (rounds 5–8) was significantly lower in the experimental group (averaged angular deviation *M* = 63.35°, *SD* = 7.45°) than in the control group (*M* = 29.62°, *SD* = 13.71°), *F*(1, 39) = 76.07, *p* < .001, ɳ_p_^2^ = 0.66. Thus, the participants in the control group performed significantly better than the experimental group in the sense that they were able to predict the next objects’ positions more precisely based on the previous pattern of displayed objects. However, the participants did not receive any explicit feedback on their performance-based payment until the end of the experiment. The participants’ age, which was included in the ANCOVA, had no significant influence on the task performance.

#### Subjective control

**Fig 3:**
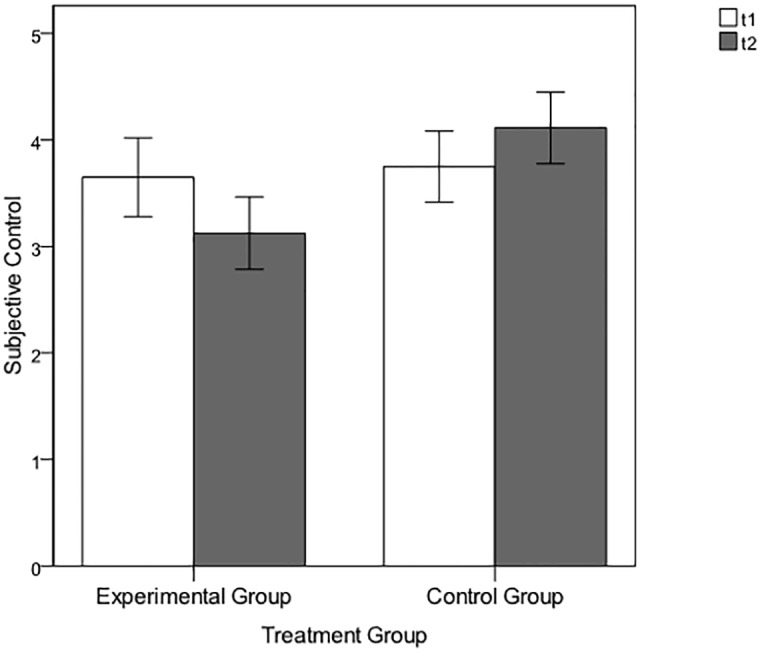
Subjective control ratings of the experimental and control group of Pilot Experimental Study 2 following the baseline (t1) and the manipulation (t2) sections. Error bars represent standard errors of the mean (±1 *SE*).

As expected, there were no significant differences in the subjective control evaluations between the treatment conditions following the baseline part at t1, EG: *M* = 3.65, *SD* = 1.66; CG: *M* = 3.75, *SD* = 1.56; *F*(1, 39) = 0.35, *p* = .556, ɳ_p_^2^ = 0.01. Also in line with our expectations, following the experimental manipulation at t2, the experimental group rated their subjective control significantly lower than the control group, *F*(1, 39) = 5.76, *p* = .021, ɳ_p_^2^ = 0.13; EG: *M* = 3.13, *SD* = 1.52 versus CG: *M* = 4.11, *SD* = 1.58 (see Fig 3). The participants’ age, which was entered as a covariate because of group differences between the EG and CG, showed no significant effect on the subjective control ratings, neither at t1 (*F*(1, 40) = 0.41, *p* = .841, ɳ_p_^2^ = 0.001) nor at t2 (*F*(1, 40) = 4.25, *p* = .046, ɳ_p_^2^ = 0.10).

### Discussion

This study’s results support the impact of increasing task difficulty within the computer-game paradigm, thus decreasing objective control, on the subjectively perceived extent of control in the expected manner. The manipulation check validated the patterns’ expected difficulty and supports their inclusion into the experimental paradigm as a manipulation of objective control. Concerning the subjective control ratings, the hypothesized feeling of loss of control induced by the difficulty-related treatment within the problem-solving game was supported. Thus, the developed experimental design based on different difficulty-level patterns of problem-solving tasks, and therefore changes in objective control, can be regarded as appropriate for inducing a subjective sense of loss of control in participants in the experimental group during the manipulation part compared to the baseline part as well as the control group.

## Main Experimental Study 1

### Aims

The objective of the first main experimental study was to prove the efficacy of the newly developed experimental paradigm via investigating the spillover effects of loss of control on risk perception. By applying the new paradigm, this study aimed at exploring the hypothesized spillover effect on subjective risk perceptions as a cognitive indicator of risky decision-making.

### Method

#### Participants and data collection

The study was conducted using a student sample consisting of *N* = 50 (50% female) participants recruited via ORSEE [37]. Their average age was *M* = 23.30 years (*SD* = 2.12, range: 19–30). There was a random assignment to the EG (*n* = 25; 48% female) and the CG (*n* = 25; 52% female). Voluntary participation was compensated by a fixed participation fee (11 €) supplemented by payment according to the subject’s performance on the problem-solving task (theoretical range: 0–8 €).

Participants were informed that the study was a two-part experiment. In the first part, Main Experimental Study 1 consisted of the same experimental computer-based problem-solving paradigm as Pilot Experimental Study 2, but instead of applying questionnaires on subjective control at t1 and t2 risk perception was measured using subjective estimations concerning a risky scenario following the manipulation section at t2 (cf. Fig 2). In the second part of the study, socio-demographic and trait variables were assessed during a separate follow-up attended by each participant within about two weeks following the experimental part of the study. Participants were compensated by 7 € (part of the total fixed participation fee 11 €) in the second part.

#### Variables and study measures

In order to investigate the hypothesized effect of subjective loss of control on subjective risk-related estimations participants’ risk perception was assessed by a case scenario of the computer-based variant of the “devil’s task” [43], as the Columbia Card Task (CCT, [44]) or the bomb risk elicitation task [45]. Specifically, we showed a circle with 23 equal sectors consisting of 22 “secure” and one unknown “devil’s” section after completing the manipulation section of the experiment. In the “devil’s task”, choosing a secure sector results in a gain of 0.10 € per sector while choosing the “devil’s” section, whose position is unknown, causes the loss of all money. This task reflects a typical risk situation with the number of fields chosen serving as the dependent variable. However, in this study participants’ actual behavioral decisions were not being investigated, but instead their risk perception was the variable of interest. Therefore, the participants were presented with a case vignette of the “devil’s task”, a picture of the circle in which seven of the 23 sectors had been selected (indicated by white color), and they were told to imagine that someone had made this choice in the “devil’s task”. Afterwards they were asked to indicate how risky they considered this choice using an eleven-point-rating-scale ranging from 0 *not at all risky* to *10 extremely risky*.

In addition to socio-demographic variables three sets of potentially relevant traits were assessed in a separate follow-up attended by participants within about two weeks after the experiment:

Nonverbal reasoning was assessed by three subtests (“number series”, “figure selection” and “cubes”; 20 items each) of a German intelligence structure test (I-S-T 2000-R, [39], English version, [40]).

For measuring general control beliefs, the German version of the IPC locus of control scale ([41], German version, [42]) was applied and subscales P and C were joined for a 16-items external control orientation subscale because of reliability concerns. The internal consistency of the IPC-scale was satisfying for our purposes (internal control orientation subscale [I]: α = .70; external control orientation subscale [P and C]: α = .60).

Participants’ traits regarding risk attitudes and behavior were assessed by the “financial decisions” subscale (consisting of four items each on “investing” and “gambling”) of the Domain-specific Risk-attitude scale (DOSPERT, [46], German version, [47]). The eight items separately assessed both risk perception and risk behavior. Internal consistency was satisfactory with α = .69 for the risk perception scale and α = .70 for the risk behavior scale.

The concluding socio-demographic questionnaire assessed participants’ sex and age and included questions on their subjects of study, mother languages and subjective ratings of their experiences with computer games.

### Results

#### Trait variables

Tests for equivalence did not reveal any significant group differences regarding the socio-demographic variables, indicators of nonverbal reasoning, general control beliefs and trait-based risk-related variables. Thus, no socio-demographic or trait variables were included as covariates in the following analyses.

#### Risk perception

A Mann-Whitney *U*-test revealed that–following the manipulation section–the members of the experimental group did not estimate the riskiness of the presented scenario higher than the members of the control group (EG: *M* = 3.84, *SD* = 3.51 vs. CG: *M* = 2.52, *SD* = 2.57), with *z* = -1.13, *p* = .258 (two tailed).

### Discussion

The result of Main Experimental Study 1 does not support Hypothesis 1, as it does not indicate significant increased perceptions of risk but only an increased tendency following the experimental manipulation of difficulty-related subjective loss of control. One possible explanation for this result might be that selecting 7 sectors from 23 sectors in the “devil’s task” is considered as a rather “safe” choice for most people. Thus, the preliminary evidence of the hypothesized effect of loss of control on risky decision-making measured via perceptions of risk as a cognitive indicator is not provided.

## Main Experimental Study 2

### Aims

The objective of the second main experimental study was to prove the efficacy of this new developed experimental paradigm via investigating the spillover effects of loss of control on risk-taking behavior. Thus, the consequences of loss of control for subsequent risk-taking behavior in an objectively unrelated setting were at issue.

### Method

#### Participants and data collection

The sample consisted of *N* = 47 (49% female) students with an average age of *M* = 23.30 years (*SD* = 2.85, range: 18–34). Again, the participants were recruited using the online recruiting system ORSEE [37] and were randomly assigned to the EG (*n* = 24; 50% female) and the CG (*n* = 23; 48% female). The voluntary participation was again compensated by a fixed participation fee (9 €) as well as additional payment according to the subject’s performance in the problem-solving and risk game (theoretical range: 0–10.20 €).

Participants were also informed that the study was a two-part experiment. In the first part, Main Experimental Study 2 applied the same experimental computer-based problem-solving paradigm as Main Experimental Study 1. However, following the experimental manipulation section at t2, behavioral effects in a risky situation were investigated (cf. Fig 2). Finally, there was a separate follow-up again for assessing socio-demographic and trait variables during an about two-week-interval after completing the experimental part of the study. Participants were also compensated by 5 € (part of the fixed participation fee 9 €) in the second part.

#### Variables and study measures

In order to assess the effects of subjective loss of control on risk behavior, participants were presented with a computer-based variant of the “devil’s task” [43–45] following the manipulation section of the problem-solving task. The “devil’s task” consisted of a circle with 23 equal sectors. One of these sectors was a “devil’s” sector, whose position was unknown to the participants, and the remaining sectors were “secure”. If only secure sectors were selected, the participant received 0.10 € per selected sector, if a devil’s sector was selected, they received nothing. The participants were allowed to decide on both the number and position of the fields they could choose. Thus, the more sectors participants selected the higher the possible payoff but also the higher the probability one could lose. Subjects were not immediately informed when they chose the “devil’s” sector. This information was given after they had completed their selection. The participants’ actual risk-related behavioral decision was investigated with the number of chosen fields serving as the dependent variable (theoretical range between 0 and 23).

Potentially relevant control variables including a set of socio-demographic and trait variables were assessed during the follow-up. The participants’ sex, age, subjects of study, mother languages and subjective experiences with computer games were assessed via questionnaires. Trait variables included (a) measures of numeral and figural nonverbal reasoning (subtests “number series”, “figure selection” and “cubes” with 20 items each of a German intelligence structure test: I-S-T 2000-R, [39]; English version, [40]), (b) measures of internal versus external locus of control (IPC locus of control scale, [41]; internal consistencies: internal control orientation subscale [I]: α = .63; external control orientation subscale [P and C]: α = .71, [42]), and (c) measures of trait-based risky decision-making (The computer-based variant of the “devil’s task” was applied again in order to assess the participants’ trait-based risk behavior, [43]; DOSPERT, [46]; German version, [47], subscale “financial decisions” consisting of “investing” and “gambling”, both with respect to risk perception and risk behavior; internal consistencies: α = .63 for risk perception; α = .74 for risk behavior).

### Results

#### Trait variables

No significant group differences occurred in tests for equivalence with respect to socio-demographic variables, indicators of nonverbal reasoning and general control beliefs. In addition, the experimental and control groups did not differ in trait-based risk-related variables, which included the DOSPERT self-report risk perception and risk behavior scales [46, 47] nor in their risk-taking behavior in Slovic’s [43] “devil’s task” at the follow-up investigation. Thus, trait and socio-demographic variables were not considered in the following analyses.

#### Risky behaviour

A Mann-Whitney *U*-test revealed that–following the manipulation section–the members of the experimental group chose significantly fewer sections in Slovic’s [43] “devil’s task” than the members of the control group (EG: *M* = 9.62, *SD* = 3.80; CG: *M* = 11.83, *SD* = 3.96) with *z* = -2.23, *p* = .026 (two tailed).

### Discussion

The results of this experimental study support our second hypothesis and indicate that participants engage in less risk-taking behavior following the experimental manipulation of subjective loss of control. Thus, the assumed spillover effect of experimentally induced subjective loss of control on risky decision-making could be demonstrated when assessing the participants’ real behavioral decisions in a concrete risk situation.

## General Discussion

Aimed at investigating the effects of prior experiences of loss of control on risky decision-making by applying a newly developed experimental paradigm, this set of four experiments provides empirical evidence for the importance of subjective control experiences when considering risk-related decision making. Additionally, the current set of studies makes an innovative methodological contribution. In two pilot studies the experimental paradigm using difficulty-based manipulations was designed and tested and its adequacy for inducing changes in subjectively perceived control was substantiated. Thus, we proposed a new experimental paradigm that overcomes some design-related problems of previous experimental settings. This paradigm induces experiences of loss of control in an incentivized setting, which meets the guidelines and ethical criteria of experimental economics and overcomes the shortage of previously used experimental designs in the psychological literature (e.g., [23–25]). Therefore, this new paradigm is suitable for investigating the impact of experimentally manipulated subjective control experiences.

In order to test the efficacy of this newly developed experimental paradigm, we also applied it to investigate the effect of subjective loss of control on risky decision-making. Risk perception and risk-taking behavior were separately measured as two indicators of risky decision-making in two experiments. Concrete risky decision-making differed significantly between the groups of participants who had differing experiences of control. Participants exposed to manipulations of loss of control showed stable risk perception but deviant risk-taking behavior in the following risky context in comparison to those who were able to maintain their personal control. Although risk perception was not significantly increased, it tended to increase (Main Experimental Study 1), and risk-taking behavior decreased following loss of control (Main Experimental Study 2). Both of these observations can be interpreted to be complementary as they point to the same direction, namely, decreased risky decision-making. Following an experience of a loss of control, people seem to be more cautious and averse towards risky situations. These findings are in line with previous studies which have empirically demonstrated increased levels of risk aversion following chronic losses (e.g., [48]).

Thus, the observed effects on risky decision-making partially supported our hypotheses and are in line with the theoretical approaches on which we based our assumptions. From the perspectives of the Learned Helplessness Theory, availability bias and the risk-as-feelings approach, the risk-related decision-making process is argued to be influenced by factors shaped by prior experiences or generally unrelated to the hazard, and thus, factors lying outside the actual concrete and objectively given decision-making criteria. In our study, the concrete risk behavior, one of the two manifest aspects of our participants’ risk-related decision-making process, was influenced by prior experiences of loss of personal control due to failure in the problem-solving task.

### Limitations and Implications

In this study we proposed a newly developed experimental paradigm for inducing subjective loss of control. We also provided evidence that prior experiences of loss of control impact risky decision-making from the behavioral but not the cognitive perspective. Additionally, despite these interesting findings, there are limitations to the current set of studies that should be taken into consideration. Furthermore, both our findings and the limitations point to implications and further developments that might be explored in future research.

First, by developing this paradigm we made an innovative methodological contribution which solves some of the design-related problems associated with previous experimental settings for investigating the effects of subjective control experiences. We have provided empirical evidence on the applicability of our experimental design through Study 2 but not through Study 1. One possible explanation might be that the example we applied to assess risk perception in Study 1 was not optimal. Selecting 7 sectors from 23 sectors is rather “safe” as compared to the nearly 12 sectors that participants had selected on average in the normal situation in Study 2. It is even safer when considering the situation without the participants themselves being involved in the uncertain context. Therefore, we further investigated the spillover effect of loss of control on the real risk-taking behavior in Study 2 by involving participants to make risky decision in a concrete risk situation. Further efforts for empirical validation of the experimental paradigm are certainly needed. For this purpose, it might be wise to increase the risky degree of the targeting context as well as participants’ involvement in the risky context when studying the spillover effect of loss of control on risk perception.

By meeting common guidelines (i.e. avoiding deception), and thus ruling out participants’ potential doubts and fostering reliable decisions, this paradigm overcomes the previous lack of adequate paradigms. For these reasons, our newly developed paradigm is able to facilitate further research on the effects of subjective loss of control which is of interest to a broad range of perspectives, such as various scientific disciplines (e.g., psychology or economics) and subsamples (e.g., students, clinical and nonclinical samples, economic decision-makers). Being characterized by a stable and highly standardized setting, and thus meeting objectivity requirements and enabling control of confounding individual and situational variables (e.g., traits, preexisting experiences with similar tasks, noise, time of day, etc.), the paradigm can easily be adapted to investigate related issues and research questions and broaden our understanding of the impact of control experiences on everyday perceptions, behavior and decision making.

In the face of the recent discussion on the observable differences between risk-related decisions that are either based on descriptions or on experiences [49, 50] we have to point out that our design is based on descriptions, thus limiting the extent to which our results may generalize. Given the fact that most of the time in everyday life people cannot rely on objective descriptions of the possible consequences of alternative options but instead make decisions based on previous experiences or feedback-based learning processes, future studies might add valuable evidence building on the present results. In particular, questions about the intensity and duration of the spillover effects of loss of control in the face of more current experiences during the decision-making setting might be answered by comparing our results gained by a description-based method with those investigated by an experience- or feedback-based method for assessing risk-related decisions. Considering the flexibility of our experimental designs, the present studies easily allow these kinds of future investigations within experience- or feedback-based decision making settings.

Furthermore, future studies should also address the mechanisms underlying the observed spillover effects. In particular, it would be helpful to identify variables that can act as a link between experienced loss of control and a risk-taking decision. These indirect or mediating effects could then help to explain how the loss of control experiences affect subsequent risk-related decision making in an objectively unrelated setting. In this context, the impact of two (groups of) variables on the association between experiences of loss of control and risky decision-making seems especially plausible. First, it is argued that prior experiences might affect subsequent risk estimations and decisions by lowered expectations of success, decreased illusionary control and vivid mental representations of losses (cf. availability bias or risk-as-feelings approach). Second, a group of variables shown to be both related to experiences of loss of control and to impact risk judgments and behavior are emotions (e.g., impact of state anxiety on risk perception, [51]; differential impact of fear and anger, [52]). Affective states have also been explicitly included in theoretical models of risk-related decision making, such as in the risk-as-feelings-approach. Considering our results, emotions following from the experimentally induced subjective loss of control experiences might act as a link between the different settings and might be able to explain the observed spillover effects (for relations between control and emotions in different domains see [53]). In this sense, taking into account possible affective states, following the experimental manipulation could potentially reveal mediating effects of emotions on the relationship of prior control experiences and risky decision-making.

### Conclusion

It is generally acknowledged that perceptions and experiences of control are a central aspect for human decision making. However, there has been no appropriate experimental paradigm for inducing experiences of loss of control in an incentivized setting. Therefore, this study contributed in developing an experimental paradigm for inducing loss of control. In order to prove the efficacy of this newly developed paradigm, we applied it with respect to investigating the spillover effects of loss of control on risky decision-making. Our results proved that it is an efficient method for manipulating subjective control experiences. Thus, it might facilitate further steps for investigating the effects of experiences of control on human decision making. In addition to the methodological contribution, this study also provided empirical evidence that experiences of loss of control impact risky decision-making when considered behaviorally but not cognitively. Understanding the processes underlying risk-related decision-making could contribute to supporting people in improving the way they deal with risky situations [54]. Based on our results it seems reasonable to focus on people’s preceding experiences, especially on their perceptions of control. Taking previous incidental control experiences into account when making a decision may be either wise or not, depending on the situation. Future research should focus on the underlying processes in more detail. Knowledge of the underlying associations may make an important contribution to support people in dealing with critical events, such as experiences of loss of control, and allowing them to master challenges, such as decision-making in the face of risky situations.

## Supporting Information

S1 Appendix RefNo: IRB15KN011ML. This document is the ethical statement of the experiment.
(PDF)

S2 Appendix Angular sequences of the 12 selected patterns. This document contains the angular sequences underlying the 12 selected patterns.
(PDF)

S1 Dataset This folder file contains the SPSS dataset of the experiment. (7Z)
