# Supplementary material for: Spillover Effects of Loss of Control on Risky Decision-Making
Source: PLoS One. 2016 Mar 1;11(3):e0150470. doi: 10.1371/journal.pone.0150470 (PMC4773176; doi:10.1371/journal.pone.0150470)
Supplement: S1 Appendix — This document is the ethical statement of the experiment. (PDF) [file pone.0150470.s001.pdf]

University of Konstanz, Box M657, D-78457 Konstanz

University of Konstanz
Department of Biology

Prof. Dr. Marcel Leist
Doerenkamp-Zbinden-Chair -
***in vitro* toxicology and biomedicine**

Phone: +49 7531 88-5037
Email: Marcel.Leist@uni-konstanz.de

Date: 03.06.2015

Specific project title. "Spillover Effects of Loss of Control on Risk Propensity"

RefNo: IRB15KN011ML

To whom it may concern,

in my function as the Head of the Ethics Committee (institutional review board, IRB) of the University of Konstanz, I would like to deliver the following statement regarding the research project "Spillover Effects of Loss of Control on Risk Propensity" submitted by Prof. Dr. Thomas Goetz, and performed in the context of the employment of the applicant at the University of Konstanz:
After vetting the Description of Work (DoW), the project has been found to fall outside the range of projects requiring an IRB statement. For this type of project, approval by the IRB or any regulatory body is not required according to our and national statutes in Germany. This study collected was non-invasive, and any other obvious issues concerning a threat to human health, well-being and dignity have not been identified.
Like for any other research project, the IRB expects this project nonetheless to be monitored by the PI for any ethical issues arising. It is within the normal scientific responsibility of the PI to decide on initiation and continuation of the project. The University of Konstanz IRB cannot relieve researchers from their basic responsibilities linked to all scientific projects at the university, but it has an administrative procedure in place that will ensure that ethics guidelines as laid down in German national law and international conventions are fully respected. The Ethics Committee (IRB) will ensure and, if necessary, enforce the proper adherence to ethics guidelines during and after the project.
This judgment was made on the basis of following information and guidelines: (1) the text of the Publication and the study outline, submitted on 28th May 2015; (2) national and international guidelines (in particular the WMA Declaration of Helsinki - Ethical Principles for Medical Research Involving Human Subjects). Recommendations by German and Swiss medical associations as well as societies of psychology have also been taken into account.

Sincerely,

Prof. Dr. M. Leist

Paketanschrift/Parcel address
Universitätsstraße 10
D-78464 Konstanz

Sekretariat/Secretary
Brigitte.Schanze@uni-konstanz.de
Tel: +49-7531-885038
FAX : +49-7531-885039
