# Supplementary material for: Spillover Effects of Loss of Control on Risky Decision-Making
Source: PLoS One. 2016 Mar 1;11(3):e0150470. doi: 10.1371/journal.pone.0150470 (PMC4773176; doi:10.1371/journal.pone.0150470)
Supplement: S2 Appendix — This document contains the angular sequences underlying the 12 selected patterns. (PDF) [file pone.0150470.s002.pdf]

Angular sequences of the 12 selected patterns

Selected Pattern	Angular sequence							
1	147							
2	30	-20	10	70				
3	10	135						
4	60	60	60	60	60	60	120	120
5	39	82	-11	79	160	12		
6	15	-30	45	-60	75	-90	180	
7	20	90						
8	45	-90	135	180	-135	90	-45	
9	20	70	-20	-70	-20	-70	20	70
10	42	84	-42	-100				
11	90	180	-90	90	90	-90	180	
12	45	180	-30	15	-180	-15	165	-90
